# Editorial: In celebration of women in cell adhesion and migration

**DOI:** 10.3389/fcell.2023.1348958

**Published:** 2023-12-11

**Authors:** Claudia Tanja Mierke

**Affiliations:** Faculty of Physics and Earth Systems Science, Peter Debye Institute of Soft Matter Physics, Biological Physics Division, Leipzig University, Leipzig, Germany

**Keywords:** mechanoresponse mechanism, matricellular proteins and extracellular matrix (ECM), ADAM8, plastin-3, nuclear localization, macrophage and dendritic cell invasion, Thy-1, adhesome

In Celebration of Women in Cell Adhesion and Migration represents a specialized Research Topic (RT) in the Section Cell Adhesion and Migration of the Journal for Frontiers in Cell and Developmental Biology Research, as it covers six excellent and fitting into the times articles, such as two review articles, one mini-review article and three original research articles. These articles cover a wide range of research fields, such as wound healing with a focus on matricellular proteins (Cárdenas-León et al.), the integrin adhesome with a special emphasis on the integrin/syndecan cell surface ligands, comprising Thy-1 (CD90) (Valdivia et al.), the mechanosensing and mechanotransduction with a special focus on cell-cell and cell-matrix adhesion complex associated proteins that translocate into the nucleus upon mechanical signals (Haage and Dhasarathy), the mechanoresponse mechanism protein, the actin-bundling protein plastin-3 (PLS-3) (Chin et al.), functional role of a disintegrin and metalloprotease 8 (ADAM8) in cell mechanics regulating cellular motility in 3D extracellular matrices (Hayn et al.), and on *Leishmania* infection that impact the motility of macrophages and dendritic cells in 3D (Luz et al.). The mechanobiology aspect of basic cellular processes such as cell adhesion and migration has increased the complexity of these processes and requires the identification of physical principles to understand the underlying mechanisms (Eyckmans et al., 2011; Mierke, 2014, 2019, 2020; Yap et al., 2018). In this context, it is critical to select an adequate microenvironment for the assessment of cell adhesion and migration, as an interaction exists between the phenotype of the cytoskeleton, the nuclear or organelle phenotype of the cells and their 3D ECM microenvironment (Kunschmann et al., 2017; Mierke, 2020; Pawluchin and Galic, 2022).

In their review article, Cárdenas-León et al. described the influence of matricellular proteins on the process of the healing of tissue after injury. Skin wound repair is a comprehensive procedure that involves changes in all parts of the skin, even the ECM. The ECM is composed of major structural proteins including collagens and elastin as well as smaller proteins with mostly regulatory characteristics, referred to as matricellular proteins. Matricellular proteins are sequestered into the ECM, and while they are able to attach to structural ECM elements like collagen fibrils or basement membranes, they are hypothesized not to participate in their mechanical actions (Bornstein, 1995, 2009; Feng et al., 2019). However, by interacting with other ECM proteins, such as fibronectin, matricellular proteins of the families of thrombospondins, tenascins, and SPARC (Morandi et al., 2023) can indirectly interfere with mechanical actions, as it has been shown for fibronectin, for example, that the mechanical signals of the cell’s microenvironment change (Aermes et al., 2020) thereby altering cell mechanics (Scott et al., 2015). The functions of matricellular proteins include the interaction with cell surface receptors, proteases or cytokines and the triggering of a cellular reaction. In opposition to the permanent occurrence of structural proteins in the ECM, the expression of matricellular proteins is strictly coordinated to fine-tune their functioning in the maintenance and damage healing of tissues (Nikoloudaki et al., 2020). The signaling triggered via matricellular proteins regulates the differentiation and proliferation of cells, which affects the regeneration of tissue. In this review, the authors present an insight into the matricellular proteins, such as cellular communication network factor family, thrombospondin family, tenascin family, fasciclin family, the secreted protein, acid and rich in cysteine family, the small integrin-binding ligand, N-linked glycoproteins family, the galectin family, the R-spondin family, the fibulin family, ecto-nucleotide pyrophosphatase/phosphodiesterase family, and the olfactomedin family which have been proven to be implicated in the process of cutaneous wound repair and consolidate the knowledge available so far regarding their respective functional roles in this process.

Cell adhesion and migration rely on the formation and breakdown of adhesion frameworks commonly referred to as focal adhesions. Cells attach to the ECM and create these structures through receptors like integrins and syndecans, which trigger signal transduction routes that couple the ECM to the cytoskeleton and thus control adhesive and migratory events. ECM proteins represent the prototypical integrin ligands, but they are far from being the exclusive ones. These other ligands comprise growth factor receptors, which control cell proliferation, the differentiation and movement of cells, as well as other cell surface receptors that facilitate cell-cell interactions (Brizzi et al., 2012; LaFoya et al., 2018). In their review article Valdivia et al. presented the role of the candidate of the latter category, such as the integrin receptor (ligand) Thy-1, also known as CD90 that was identified by the same group over 22 years ago (Leyton et al., 2001). Thy-1 acts as a cell adhesion molecule comprising a singular Arg-Gly-Asp (RGD)-like peptide that engages and stimulates various integrins to encourage cell-to-cell exchange. Thy-1 also possesses a heparin-binding domain (HBD) for activating syndecan-4, which is an actor of equal relevance in the process of cell migration (Valdivia et al., 2020). The important point is that the findings by the authors have highlighted two players in the integrin adhesome, such as on the one hand Thy-1 that functions as a receptor for integrins at the cellular level (Leyton et al., 2001; Zaidel-Bar et al., 2007; Kanchanawong and Calderwood, 2023), and on the other hand, the cell polarization protein Partition-defective 3 (PAR-3) that tethers to non-integrins (Valdivia et al., 2020). The authors originally characterized Thy-1 as a tethering cognate of integrin β 3 (Leyton et al., 2001), and later Thy-1 was classified as an α v β 3 integrin adhesome cognate compound (Zaidel-Bar et al., 2007), while PAR-3 was found to interfere with syndecan-4 to induce phosphorylation of FAK (Valdivia et al., 2020). Thy-1 is therefore able to engage and activate integrins and syndecan-4 at the same time to convey mechanotransduction. Cell-to-cell interactions (in trans) or within the same cells (in cis) can cause this multi-receptor interference. Integrins attach to the ECM and soluble or cell surface ligands to build integrin adhesion complexes (IAC) that vary in their composition according to cellular environment and specific cell type. Proteomics analyses of these IACs resulted in the establishment of the concept of the adhesome, which refers to a complicated molecular framework of hundreds of proteins participating in signal transmission, adherence and locomotion of cells. A hallmark of these IACs is the perception of mechanical signals generated by the stiffness of the ECM and the tension imposed by cell-cell interfaces, and the transmission of this force by altering the actin cytoskeleton to govern the migration of cells. The authors have described Thy-1 (CD90) as an integrin/syndecan cell surface ligand, which is a GPI-anchored protein that has specific engagement domains for these receptors and, when in contact with them, promotes the adhesion and migration of cells. In their detailed review, the authors address what is presently understood of adhesomes, consider how mechanical forces have altered the perspective on the way cell migration is governed, and comment on how the authors’ group has advanced knowledge of the cell signaling mechanisms that regulate cell adhesion and migration.

In their mini-review article, Haage and Dhasarathy focus on the mechanism of mechanosensing and mechanotransduction processes and highlight the involved molecules that are mostly well-known proteins acting on two sites within the cells. Cells are able to perceive alterations in their surroundings, transmit signals internally so that they can respond to external cues in a controlled manner and thereby trigger adaptive alterations in the state and performance of the cells. Frequently, this reaction entails modification of gene expression within the cell nucleus, which is widely viewed as a physically distinct process from the remainder of the cell. Proteins that conventionally serve as a sticky glue at the cell membrane, which aids cells to attach to one another or to the ECM, have been recognized in new localization schemes inside the cell nucleus. These include proteins, such as Zyxin, FAK, T-cadherin, β -catenin, Zonula Occludens-1 (ZO-1) and Zonula Occludens-2 (ZO-2) as well as plakofilin and plakoglobin, which constitute adherens junctions, desmosomes, focal adhesions, gap junctions and tight junctions. It is more and more accepted that these cell adhesion proteins, which are usually involved in either signal uptake and/or short-term reactions, are also distributed in the cell nucleus and are therefore directly implicated in the regulation of genes, which constitutes a traditional long-term reaction, with no requirement for intermediate signal transmitters (Hervy et al., 2006; Zuleger et al., 2012; Zheng and Jiang, 2021). The authors stated that this finding further unravels the partitioning of work between molecules that operate on relatively short- and long-time scales. Several macromolecular structures are implicated in the process of mechanosensing, in which a cell perceives physical cues and incorporates and transduces these cues into downstream reactions like signal transduction cascades and alterations in gene regulation. The ECM and the macromolecules that compose it are an integral component of the cellular microenvironment. They allow cells to engage with one another and deliver both structural and biochemical cues that are perceived via transmembrane adhesion receptors. This avenue of communication between the ECM, the cell adhesion proteins and the cytoskeleton governs cellular performance, the dysfunction of that behavior leads to disease. New insights indicate that several of these adhesion proteins have more direct functions in chromatin structure and gene regulation, RNA maturation and other non-canonical activities. Although most of these findings were restricted to monitoring cytoplasmic-nuclear trafficking, recent progress in microscopy, biochemistry, proteomics and genomics has considerably enhanced the knowledge of the consequences of nuclear localization of these proteins. In their review article, the authors look closely at well-known cell adhesion proteins that translocate into the cell nucleus and their subsequent activities. The authors discuss the most recent progress in this fascinating but still young discipline, with implications that span from fundamental biology to disorders such as cancer.

In their original research article, Chin et al. focused on the function of plastin-3 (PLS3) as being part of the mechanoresponsive machinery. PLS3 represents a calcium-sensitive actin binding protein that has just been implicated in the pathogenesis of infant osteoporosis. There is clinical evidence to propose that PLS3 mutations result in compromised osteoblast functionality, although the underpinning mechanism is still elusive. The authors created MC3T3-E1 preosteoblast cells stably deficient in PLS3 to determine the involvement of PLS3 in the mineralization of bone. The authors were able to demonstrate that after analysis of the osteogenic differentiation of PLS3 knockdown cells (PLS3 KD), the first phase of osteoblast mineralization, during which a collagen matrix is sequestered, is not disturbed, but the consecutive mineralization of this matrix is strongly impaired. At this stage, osteoblasts depend strongly on mechanosensitive signaling circuits to maintain mineral accumulation as a reaction to the growing ECM stiffness. PLS3 is mainly visualized at focal adhesions, which are closely associated with mechanosensitivity. Consistent with this, the authors revealed that depletion of PLS3 resulted in osteoblasts not reacting to alterations in ECM stiffness and exhibiting the equal cell size, length of focal adhesions and number of focal adhesions when seeded on soft (6 kPa) and stiff (100 kPa) media, unlike control cells where each parameter increased when seeded on 100 kPa media. Failure of PLS3-KD cells to expand on stiff substrates was rescued when wild-type PLS3 was expressed, whereas expression of three PLS3 mutations identified in patients with early-onset osteoporosis with abnormal actin-binding activity failed to rescue this defect. Finally, the authors demonstrate that actin bundling by PLS3 is involved in the mechanosensitive process that enhances mineralization of osteoblasts.

Most cancer cell studies were conducted in a highly simplified 2D *in vitro* microenvironment. Over the last 10 years, there has certainly been a tendency for more sophisticated 3D *in vitro* cell culture systems that can overcome the current gap between 2D *in vitro* and *in vivo* experiments in the field of biophysical and cell biological cancer cell research. Hayn et al. proposed the hypothesis that the bidirectional interplay between breast cancer cells and their surrounding cancer microenvironment is crucial for the fate of the disease (Mierke, 2023). The tissue reorganization processes triggered by cancer cells are relevant for the mechanical testing of their matrix surroundings controlled by cancer cells and for the adhesion and movement of cancer cells. In researching remodeling processes, the focus was on matrix metalloproteinases and not on ADAMs. Nevertheless, the regulatory function of ADAM8 in cell mechanics, which controls cellular locomotion in 3D collagen matrices, is poorly understood. In their original research article, the authors therefore concentrate on the function of ADAM8 in matrix rearrangement and in the migration of extracellular 3D matrix architectures. Consequently, human MDA-MB-231 breast cancer cells in which ADAM8 was knocked out (ADAM8-KD cells) and scrambled MDA-MB-231 control cells (ADAM8-Ctrl cells) were characterized for their ability to engage and translocate in dense 3D ECMs. The fiber displacements, i.e., the ability of the cells to displace the ambient 3D matrix scaffold, were monitored. ADAM8-KD cells dislodged the collagen fibers more effectively in comparison to ADAM8-Ctrl cells. In addition, ADAM8-KD cells migrated into 3D collagen matrices in greater quantities than ADAM8-Ctrl cells. The inhibition of ADAM8 by the ADAM8 inhibitor BK-1361 resulted in markedly elevated fiber displacements of ADAM8-Ctrl cells to the same level as ADAM8-KD cells. On the contrary, the inhibitor failed to affect ADAM8-KD cells in terms of fiber displacements and quantitative features of cell invasion of ADAM8-Ctrl cells, although the cells detected in the matrix penetrated significantly deeper. Fiber displacements of both cell types accelerate when matrix restructuring by cells is disrupted by a broad-spectrum metalloproteinase inhibitor, GM6001. It is generally acknowledged that ADAM8 breaks down fibronectin in a direct and/or indirect fashion. The addition of fibronectin prior to polymerization of the 3D collagen matrices led to an increase in fiber displacements and cell invasion in fibronectin collagen matrices of ADAM8-Ctrl cells, while the fiber displacements of ADAM8-KD cells remained the same. The addition of fibrinogen and laminin, by contrast, led to an augmentation of fiber displacement in both types of cells. The authors concluded that the influence of fibronectin on the selective enhancement of fiber displacement of ADAM8 Ctrl cells appears to be ADAM8-dependent. ADAM8’s presence could therefore explain the long-disputed findings of fibronectin accumulation in the malignant development of cancers like breast cancer. In conclusion, ADAM8 appears to be fundamental for cell-driven fiber displacements of the ECM environment that increases 3D cell movement in a fibronectin-rich milieu.

In their original research article, Luz et al. point out that leishmaniasis causes a widespread spectrum of clinical manifestations, extending from skin lesions at the point of infection to more widespread lesions in internal organs like the spleen and liver. Whereas the capacity of *Leishmania*-infected host cells to undergo migration may be relevant for lesion dissemination and parasite spread, the governing mechanisms and the accompanying involvement of host cells continue to be scarcely characterized. *Leishmania* infections have been implicated in impairing macrophage migration in a two-dimensional setting through changing actin dynamics and affecting the expression of proteins participating in the interplay between plasma membrane and ECM. Even though *L. infantum* has been proven to promote dendritic cell migration in 2D, cell migration *in vivo* mainly takes place in a three-dimensional (3D) environment. The authors’ aim was to study the movement of macrophages and dendritic cells infected with *Leishmania* in a 3D model and to elucidate the mechanisms underlying this movement. Cell migration, adhesion complex generation and actin polymerization were evaluated following infection of mouse bone marrow-derived macrophages (BMDM), human macrophages and human dendritic cells by *L. amazonensis*, *L. braziliensis* or *L. infantum*. The authors revealed that *Leishmania* infection blocks 3D migration in BMDM and human macrophages. Decreased expression of proteins implicated in adhesion complex generation and variations in actin dynamics were also detected in *Leishmania*-infected macrophages. Conversely, the enhanced movement of human dendritic cells in a 3D setting has been associated with enhanced adhesion complex generation and heightened actin dynamics. In summary, the authors’ findings indicate that *Leishmania* infection hinders the 3D migration of macrophages whereas it promotes the 3D migration of dendrites by altering actin dynamics and the expression of proteins that participate in the interface between plasma membrane and ECM, pointing to a probable connection between dendritic cells and the visceralization of the disease.

All articles of the RT show the great diversity in the field of cell adhesion and migration analysis. In particular, reference is made to the mechanical aspect of these processes, which has so far been completely ignored in these special fields of research. Mechanobiology is increasingly finding its way into classical cell biology and molecular biology research. The importance of cell surface receptors such as integrins; syndecans and ADAMs, such as ADAM8, cell-matrix receptor associated proteins on the cytoplasmic face and cytoskeletal tethering proteins, such as PLS3, and the ligand binding of matricellular proteins to cell surface receptors, cytokines or proteases has been emphasized in processes, such as mechanosensing and mechanotransduction. Finally, there are many more interesting facts to explore in this regard, e.g., the question of the interaction of matrix cellular proteins and ECM proteins such as fibronectin and their influence on the mechanical properties of the cell and the translocation of cytoskeletal focal adhesion proteins into the nucleus and their new function there. Everything could shed more light on these complex processes to better understand physiological processes and pathological developments.
